# Medical students’ perceptions of AI-based feedback and feedforward on communication skills in doctor–patient consultation - an acceptance study in a video-based simulation

**DOI:** 10.1080/10872981.2025.2592414

**Published:** 2025-12-01

**Authors:** Moritz Bauermann, Thomas Rotthoff, Tobias Hallmen, Miriam Kunz, Elisabeth André, Ann-Kathrin Schindler

**Affiliations:** a Medical Didactics and Education Research, DEMEDA, Faculty of Medicine, University of Augsburg, Augsburg, Germany; b Human-Centered Artificial Intelligence, Faculty of Applied Computer Science, University of Augsburg, Augsburg, Germany; c Medical Psychology and Sociology, Faculty of Medicine, University of Augsburg, Augsburg, Germany

**Keywords:** Artificial Intelligence, avatar, feedback, feedforward, health communication

## Abstract

Feedback and feedforward are highly relevant in promoting students’ learning. With advances in artificial intelligence (AI), new opportunities to support feedback and feedforward are emerging. However, few studies have explored how medical students perceive and accept AI-based feedback and feedforward in medical communication training. In this study, we explored medical students’ perceptions of AI-based and avatar-mediated feedback and feedforward in a simulation applying a doctor–patient consultation video. The participants comprised 82 medical students (56.1% female), 66 of whom were in their second semester and 16 in their fourth semester. Before participants saw a video of a medical student in a standardized pre-recorded consultation, they were asked to put themselves in the position of the peer shown. A human-like avatar subsequently provided AI-based feedback and feedforward for the medical student in the video. The participants—still taking on the shown medical student’s role—were then asked to rate the perceived trustworthiness and their potential learning acceptance of the AI-based feedback and feedforward. The participants’ ratings of trustworthiness and potential learning acceptance were higher for the AI-based, avatar-mediated feedforward than the feedback. Additionally, they reported a generally positive attitude toward AI. This attitude was positively correlated with a higher potential learning acceptance of feedback. The tendency to favor feedforward over feedback in interpersonal contexts—as described in the literature—was evident for the perception of the AI-based, avatar-mediated evaluations of a simulated doctor-patient consultation video. Future research could apply these insights to enhance AI-based learning in medical education, e.g. by providing students with AI-based feedforward on their own consultation videos and assessing their perceptions of the same.

## Introduction

Medical students can develop their communication skills in simulated patient programs, where actors play the role of patients and students act as medical students on internship or doctors. The realism of these simulated doctor–patient consultations has been shown to effectively support medical students in developing communication and relationship skills [1,2]. However, assessing medical students’ performance in simulated doctor-patient consultation often requires significant resources and lacks scalability [3,4]. Artificial intelligence (AI) can assist by identifying and reporting specific communication signals, such as turn-taking, tone of voice, semantics, and facial expressions [5]. When this AI-generated data is disclosed to people, their acceptance of the data may be dependent on multiple aspects:

First, the *content* of the presented AI-generated data can have an impact on how it is perceived [6–8]. Content can be conveyed as feedback (retrospective perspective) or feedforward (prospective perspective) [9]. Feedforward is considered more effective from learners’ perspectives and given the potential for improved learning outcomes [10,11], yet it is used less commonly [12]. Both need to a) be trusted [13,14] and b) be accepted as useful for one’s own learning [7,15]. This learning acceptance can be viewed as a precursor and prerequisite of learning effectiveness, in which the success of learning is measured through evidence-based analyzes of changes in behavior or knowledge [16,17].

Second the *medium* presenting the AI-generated data can have an impact on its acceptance [18,19]. Findings by Weitz et al. [19] indicated that avatars are a more effective *medium* for the presentation of AI-generated data than text or video. Furthermore, the avatar’s perceived sympathy, a positive emotional affection and goodwill towards another person [20,21], has been shown to be beneficial [22,23]. Regarding the appearance of avatars in first contact situations, generalized studies found young female faces to be most trustworthy [24].

Third, AI as a *source* must be perceived as credible [7,8,25]. This credibility rests on the reliance of the presented data [26,27]. For AI-generated data to be considered reliable, it needs to align with the context in which it is applied and responsive to the specific needs of its users [28–30]. Therefore, individual digital literacy and attitudes toward AI can have an impact on users’ adoption and perceptions of its data [31–34].

**Figure 1.**
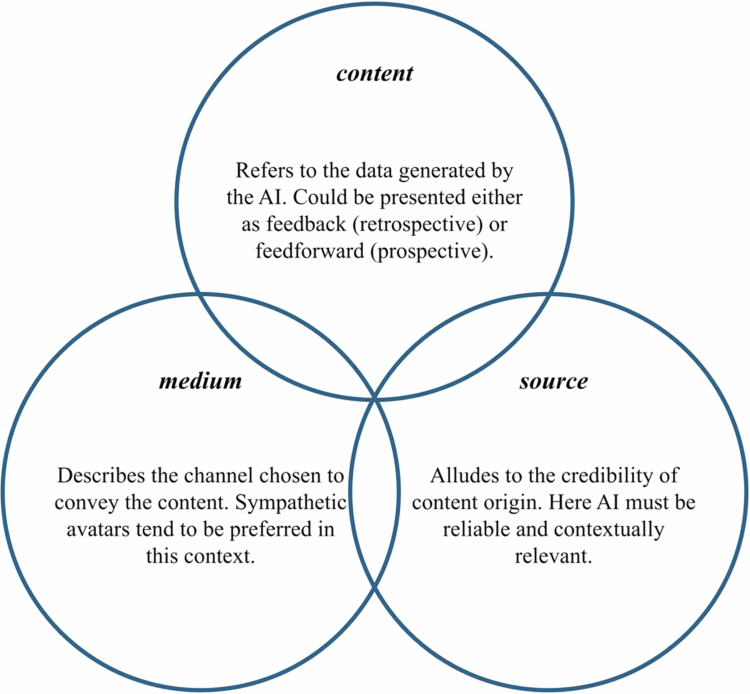
Facets of AI in medical education. Note: Original illustration.

The described interplay of *content*, *medium*, and *source* is illustrated in Figure 1, which serves as the theoretical rationale for this study, its research questions, and the findings presented.

The adoption of AI for communication skills training in medical education is already progressing [35,36]. However, a fully automated AI-generated feedback and feedforward solution for simulated doctor–patient consultations that integrates *content*, *medium*, and *source* for medical students on their own simulated doctor-patient consultations was at the time of the study absent. Moreover, little is known about medical students’ trust in and learning acceptance of AI-based feedback and feedforward for communication training.

The following Research Questions (RQ) were examined:

RQ 1 on *content*: How do medical students perceive AI-based feedback and feedforward on a videotaped simulated doctor-patient communication performed by a peer in terms of

a) trustworthiness and
b) acceptance for learning purposes?

RQ2 on *medium*: How is medical students’ perception of an avatar in terms of

a) sympathy
b) human-likeness, and
c) trustworthiness

associated with their trustworthiness and potential learning acceptance in the avatar’s communicated AI-based feedback and feedforward?

RQ3 on *source*: How are medical students’ attitudes toward AI associated with

a) trustworthiness and
b) potential learning acceptance of the AI-based feedback and feedforward?

## Methodes

### Sample

We recruited 82 medical students (56.1% female) who were enrolled in a new integrated, competency-based medical curriculum^1^ at the University of Augsburg. Among them, 66 students were in their second semester and 16 in their fourth semester. In such a curriculum, students receive simulated doctor–patient consultation communication training within their first year of study, and the study participants were therefore familiar with the setting [37].

### Design

**Figure 2.**
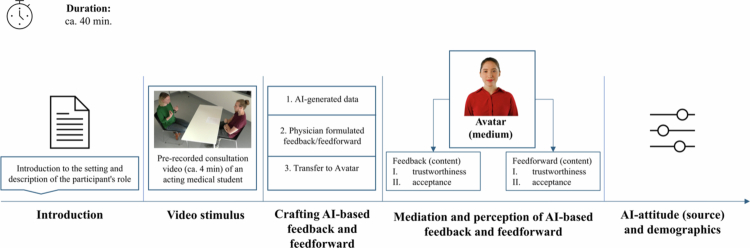
Study design. Note: Original illustration; Avatar picture via HeyGen [39].

The study was voted ethically sound by the ethics committee of LMU Munich (23−0989). The simulation took place on a 27" monitor with a 1440p resolution utilizing soSci Survey [38] providing a mouse and keyboard as interaction tools. The data was collected onsite with a standardized introduction by a research assistant, in accordance with the study design, as illustrated in Figure 2.

**Introduction.** The participants first provided their written informed consent and were then instructed to put themselves in the place of a peer who was playing a medical student in the following simulated doctor–patient consultation video with a simulated patient concerned about a tick bite. This call to take on the role of the peer was emphasized by the study instructor and in written form within the simulation (*“Please put yourself in the position of your peer shown in the video”*). Participants were provided with the learning goals of their peer in the video to further strengthen the role assumption. Lastly, they were informed that the subsequent avatar-mediated feedback and feedforward impulses were fully generated by AI, based on the previously presented consultation video. To lay the groundwork for the future use of avatars as mediators of AI in students’ own consultation videos, this preliminary study employs a controlled video stimulus that enables a systematic exploration of reactions to AI-based feedback compared to feedforward. Within higher education video stimuli, when properly used [40], have been shown to enhance participants’ emotional engagement [41]. Additionally, a video stimulus enables a controlled study setting while avoiding participants’ need to act in real simulated doctor–patient consultation processes [42].

**Video stimulus.** Next, participants watched a 4-minute video, especially produced for the purpose of this study. The recording was undertaken in a simulated consulting room of the faculty’s skills lab. The scripts for the communication scenario were developed with physicians and those responsible for the communication curriculum. The video production was supervised by an experienced physician. The role of the patient was played by a professional actor, specifically trained for this occasion and following the beforementioned script. The acting medical student was in a primary care practice and conducted a consultation with an agitated patient who had removed a tick herself and is now concerned about possible consequences. The acting medical student then had to explain that no further intervention, besides monitoring the tick bite site, was needed and had to address any further questions from the patient empathetically to ease her concerns. In doing this, the acting medical student should demonstrate a supportive, interpersonal, participatory problem-solving and transparent communication with the simulated patient [43]. Lastly, the medical student acting in the video was instructed to make some obvious mistakes regarding nonverbal communication behavior during the encounter (e.g., look at his watch often and demonstrate little eye contact with the patient) [44,45].

**Crafting AI-based feedback and feedforward (content)**. After watching the described video stimulus, participants were asked to remain in the role of the medical student featured in the video. Next, they were instructed that in this role they would now receive AI-generated feedback and feedforward on their communication skills. Both had been prepared in advance using a 3-step process. First, the video was analyzed using NOVA, an open-source video-based AI annotation tool that allows to extract selected AI-based features for conversation analysis [46]. For this analysis, NOVA solely relies on video recordings of a person’s face and body and uses a combination of existing AI models to extract eye movements and facial expressions [47]. In its current status, NOVA can generate quantified feedback from statistical behavior analyzes [48], but not in a written or spoken form suitable for learners; nor can NOVA currently provide recommendations. The quantified AI-generated data utilized for this study can be viewed in Appendix A. Second, since this study focused on the perception of feedback compared to feedforward−as *content* in a suitable didactical presentation−an experienced physician used NOVA’s AI-generated data to formulate a text for feedback and feedforward (see Appendix A). Both were tailored to the communication behavior of the medical student in the video, focusing on nonverbal communication skills such as eye contact and facial expressions [44,45]. Third the feedback and feedforward texts were then transferred to an AI powered speaking avatar using HeyGen [39]. Consequently, when we refer to *AI-based feedback and feedforward,* we mean the elaborated process whereby AI-generated data generated by NOVA were textualized by a physician and transferred to an AI-avatar.

Participants in this acceptance study were initially unaware that the feedback and feedforward had been developed through this partly manual 3-step process. This was to prevent any potential bias in investigating the acceptance and trustworthiness of fully AI-generated feedback and feedforward expected in the future. At the end of the study, participants were fully informed about the process.

**Mediation and perception of AI-based feedback and feedforward**. Consequently, from the participants’ perspective, immediately after watching the video, the avatar verbally mediated both the

- AI-based feedback (e.g. “*You sought eye contact with your counterpart about 90% of the time during the entire conversation, with the proportion being 40% in the first two minutes.*”) and
- AI-based feedforward (e.g. *“With regard to eye contact, direct eye contact with your counterpart is recommended, especially when opening a conversation. This signals interest and conveys a feeling of appreciation.”*), in a human-like behavior.

Participants were then asked to rate

- the perceived trustworthiness (*“How trustworthy did you find the measured values/recommendations for action generated by the AI regarding the communication behavior?”)* and
- potential learning acceptance (*“If you were the student in the video, would you be prepared to change your communication behaviors based on the AI-generated measured values/recommendations for action?”*)

for both the feedback and feedforward using a 6-point Likert scale (1 = not at all; 6 = fully and completely). Lastly, the participants were given the opportunity to answer an open-ended textbox question. They were asked, in their capacity as the medical student in the video, what they would have done differently next time and to explain their reasoning.

**Avatar perception (medium)**. Following this, the study participants were asked how

- sympathetic (*“How sympathetic did you find the person who provided the feedback?”)*,
- human-like (*“How natural and human-like did you perceive the AI-generated avatar to be?”)*, and
- trustworthy (*“How trustworthy did you find the human-simulated mediation of the given feedback?”)*

they deemed the avatar on a 6-point Likert scale (1 = not at all; 6 = fully and completely).

**AI attitude (source).** The participants’ attitudes toward AI were surveyed using the established German version of the ATTARI−12 [32], a 5-point Likert scale (1 = strongly disagree; 5 = strongly agree). The scale consists of 12 items addressing the three accepted facets of attitude, with four items each: cognitive (e.g., *“AI will make this world a better place”)*, affective (e.g., *“When I think about AI, I have mostly positive feelings.”)*, and behavioral (e.g., *“I would rather choose a technology with AI than one without it”)* [32,49,50]. For the later analysis, the AI attitude was treated as a single scale including the 12 items−with a reported excellent internal consistency, Cronbach’s *α* = 0.93 [32]—as recommended by the authors [32] of ATTARI−12.

**Demographic information.** Finally, the participants provided their demographic information (gender, age, and semester).

### Analysis

Analysis was conducted in IBM SPSS Statistics for Windows [51].

Due to skewed normal distribution, RQ1 was investigated using Wilcoxon signed-rank tests. Cohen’s *d* was the reported effect size, with small = .10, medium = .30, and large = .50 [52]. We tested whether participants would perceive a) trustworthiness and b) learning acceptance differently for AI-based feedback and feedforward (independent variable). a) and b) were treated as separate dependent variables.

RQ2 was investigated using Spearman’s rank correlation test *r*_*s*_ for associations of medical students’ perception of the avatar’s a) sympathy, b) human-likeness and c) trustworthiness with their perceptions of trustworthiness and potential learning acceptance of the AI-based feedback and feedforward.

Similarly, Spearman’s rank correlation test was used to explore RQ3. We tested whether a) trustworthiness and b) learning acceptance of feedback and feedforward correlate with medical students’ AI attitude. For RQ2 and RQ3 the correlation effect *r*_*s*_ was classified based on Cohen, with small = .10, medium = .30 and large = .50 [53].

Lastly, responses to the open-ended text box questions were gathered and analyzed in a thematic analysis following Braun and Clarke [54] utilizing MAXQDA [55]. The qualitative data already contained an initial thematic sorting, as the text boxes were assessed each after the trustworthiness ratings as well as to the avatar. Answers were coded deductively whether they addressed *content* (feedback and feedforward), *source* or *medium*. Next, we organized the responses within these themes, identifying clusters of similar answers and subsequently developing inductive categories. In the Results section, an exemplary selection of these categories are presented and illustrative quotes are provided.

## Results

### Medical students’ perception of AI-based feedback and feedforward (content)

The medical students’ ratings of the trustworthiness of the feedback varied from 2 to 6, with a median of 4.0 (IQR = 1.0). They rated the trustworthiness of the feedforward from 2 to 6, with a median of 5.0 (IQR = 1.0). The ratings for their potential learning acceptance of the feedback ranged between 2 and 6, with a median of 4.0 (IQR = 1.0), while their potential learning acceptance of the feedforward scored between 1 and 6, with a median of 5.0 (IQR = 1.0).

**Trustworthiness**

**Figure 3.**
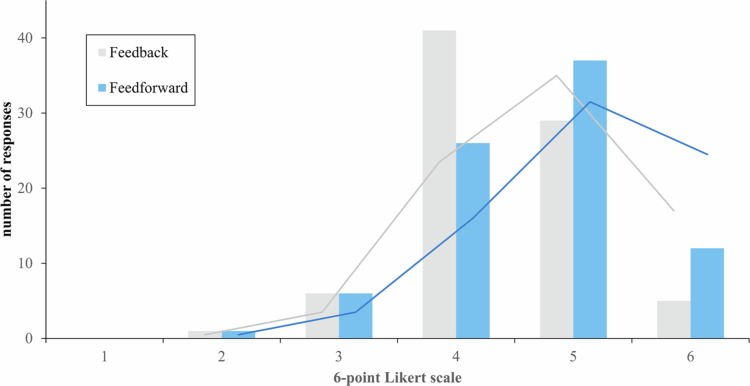
Distribution of the perceived trustworthiness of the AI-based feedback and feedforward. Note: Original illustration; 6-point Likert scale (1 = not at all; 6 = fully and completely).

RQ1a: The perceived trustworthiness of the recommended feedforward actions (median = 5.0; IQR = 1.0) was significantly greater than that of the feedback but with a small effect size (median = 4.0; IQR = 1.0; *z* = −2.745; *p* = .003; *d* = .21). This difference is visible in the comparison between the distributions shown in Figure 3.

**Potential learning acceptance**

**Figure 4.**
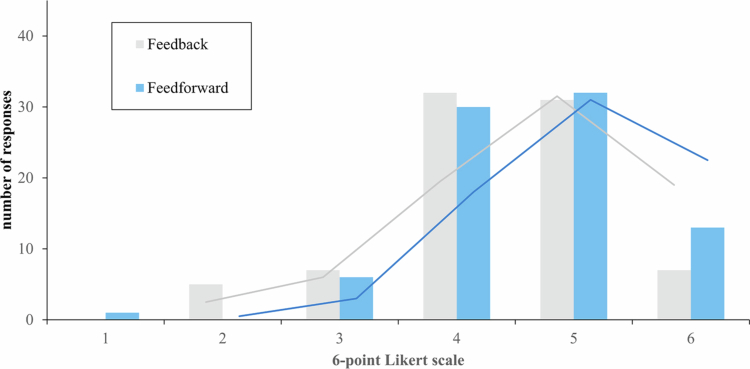
Distribution of the potential learning acceptance of the AI-based feedback and feedforward. Note: Original illustration; 6-point Likert scale (1 = not at all; 6 = fully and completely).

RQ1b: The potential learning acceptance of the recommended feedforward actions (median = 5.0; IQR = 1.0) was significantly higher than that of the feedback but with an even smaller effect size than before (median = 4.0; IQR = 1.0; *z* = −2.109; *p* = .018; *d* = .19). This difference is also visible in the comparison between the distributions shown in Figure 4.

The reported differences between the feedback and feedforward as *content* with respect to trustworthiness and learning acceptance were reflected in the open-ended textbox answers. Evidently some participants already saw room for improvement. Feedback content category: *change*:

*“Make more eye contact right from the start. The AI stated that eye contact in the initial phase of the conversation was only 40%. I would change this.”* Code 374

In contrast, others criticized in part the purely quantitative nature of the feedback provided. Feedback content category: *only numbers*

*“The feedback from the AI was very statistics-heavy and [there was] little feedback as to whether it was good or bad.”* Code 429

*“The AI has mainly given percentages, so somehow you can't do much with them. No suggested solution or evaluation is provided.”* Code 435

The feedforward recommendations, however, were received positively, and a willingness to implement them was evident. Feedforward content category: *realize advice*

*“The AI recommends eye contact at the beginning of the conversation. I found the recommendations given to be both appropriate and accurate.”* Code 366

*“Make eye contact and try to smile a little more when the situation is appropriate. The AI explained it nicely and backed it up with facts [so] that the suggested actions were justified.”* Code 368

*“The AI’s suggestions for improvement seemed understandable to me and in the interests of the patient, so I would definitely adopt something there.”* Code 399

*“I [will] try to embellish my communication with the sentence beginnings recommended by the AI. I found that the two examples very understandable and good for relationship-building.”* Code 431

### Perceptions of the avatar (medium)

**Figure 5.**
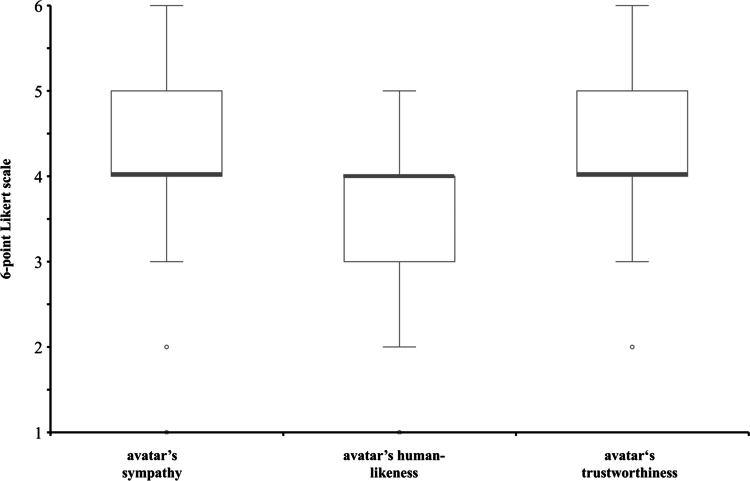
Overview of the perceptions of the avatar. Note: Original illustration; 6-point Likert scale (1 = not at all; 6 = fully and completely).

The participants’ assessments of the avatar’s sympathy varied from 1 to 6, with a median of 4.0 (IQR = 1.0), and its human-likeness was rated between 1 and 5, with a median of 4.0 (IQR = 1.0). The avatar’s trustworthiness was between 2 and 6, with a median of 4.0 (IQR = 1.0). An overview of the variables for the avatar is provided in Figure 5.

RQ2a: The participants who perceived the avatar to be sympathetic also tended to rate its trustworthiness for both feedback (*r*_*s*_ = .284; *p* = .010) and feedforward (*r*_*s*_ = .357; *p* = .001) positively. The avatar’s perceived sympathy showed a weak correlation with the potential learning acceptance of the feedback (*r*_*s*_ = .211; *p* = .057). However, the avatar’s sympathy correlated with a higher potential learning acceptance of the feedforward (*r*_*s*_ = .393; *p* = .001).

RQ2b: The participants who had a positive view of the avatar’s human-likeness also tended to rate the trustworthiness of the feedback (*r*_*s*_ = .345; *p* = .001) and feedforward (*r*_*s*_ = .243; *p* = .028) more positively. Similarly, a positive perception of the avatar’s human-likeness was associated with higher potential learning acceptance, weakly for feedback (*r*_*s*_ = .239; *p* = .030) and a little stronger for feedforward (*r*_*s*_ = .308; *p* = .005).

RQ2c: A positive rating of the trustworthiness of the human-simulated mediation was significantly linked to higher trustworthiness ratings for the feedback (*r*_*s*_ = .629; *p* = .001) and feedforward (*r*_*s*_ = .399; *p* = .001). Similarly, the participants who perceived the human-simulated mediation to be trustworthy tended to show higher potential learning acceptance of both the feedback (*r*_*s*_ = .447; *p* = .001) and feedforward (*r*_*s*_ = .490; *p* = .001).

The rather positive perception of the avatar as a *medium* for AI-generated data was also evident in the open-ended answers. Avatar medium category: *avatar approval*

*“I find it a bit unusual but definitely more trustworthy than, for example, just reading AI-generated text related to my behavior.”* Code 581

*“The human communication of the feedback seemed very real, and it made it easier to imagine and accept the constructive criticism.”* Code 555

### AI attitudes (source)

Participants AI attitude ranged from 1.58 to 4.67 with a median of 3.58 (IQR = .79). The overall Cronbach’s alpha was good (*α* = .87) [56].

RQ3a: No significant relationship emerged between overall AI attitude and the perceived trustworthiness of the feedback (*r*_*s*_ = .091; *p* = .417) or between AI attitude and the perceived trustworthiness of the feedforward (*r*_*s*_ = .127; *p* = .257).

RQ3b: The participants with a more positive AI attitude tended to report higher potential learning acceptance of the feedback (*r*_*s*_ = .256; *p* = .02). No such association was evident between AI attitude and the potential learning acceptance of the feedforward (*r*_*s*_ = .050; *p* = .658).

A positive assessment of the AI-generated data as the *source* was also found for the open textbox responses AI source category: *AI-Optimists*

*“I think the AI may be able to analyze data and figures, such as the percentage of eye contact, more precisely and provide good feedback based on this. I think it is at least a good addition to the feedback from a human.”* Code 374

*“Benefits from precise analysis and save lecturers time.”* Code 423

## Discussion

We investigated medical students’ perceptions of trustworthiness and their potential learning acceptance of AI-based feedback and feedforward for a pre-recorded simulated doctor–patient consultation video of an acting medical student. This approach provides a valuable foundation for the advancements towards a fully automated AI-generated solution, utilizing NOVA and integrating *content*, *medium* and *source* for future training scenarios with students.

Regarding *content*, the results indicated that the AI-based feedback and feedforward were generally perceived as rather trustworthy and had a relatively high potential for learning acceptance. The feedforward received significantly higher scores compared to the feedback for both trustworthiness and learning acceptance. Brooks et al. [12] argued that the forward-looking suggestions of a feedforward are better received by students and have a potentially higher impact on them. Another study of students’ perceptions of feedforward supports this claim, which suggests that effective feedforward should clarify where to go next and how to improve and provide examples [57]. Furthermore, highly informative feedforward that includes suggestions for potential next steps has been found to be very effective and most beneficial for students [58]. A recent study also indicated that AI-generated feedforward in group learning sessions may boost learning acceptance among students [59]. Based on these findings, the better adoption of the feedforward vis-à-vis the feedback as *content* observed in this study was to be expected and was corroborated.

In terms of the *medium*, the positively skewed assessments regarding the avatar sympathy, human-likeness, and trustworthiness along with positive correlations with the trustworthiness and potential learning acceptance of the feedback and feedforward indicated broad acceptance for AI-based learning environments among the study participants. In particular, the strong correlation between the perceived trustworthiness of the avatar *medium* and that of the feedback and feedforward suggests significant potential in designing an avatar that is as trustworthy as possible. Research already is exploring avatar individualization for this matter [60,61]. Generally, the use cases of avatars in these environments and their acceptance are currently growing by the day [62–65]. Similarly, the rising popularity of AI in higher education and medicine is well documented [30,66,67]. It can therefore be reasonably assumed that AI-generated avatars in digital learning environments may have an impact on the future of medical education.

Lastly for the *source*, we found a significant correlation between the AI attitude ratings and potential learning acceptance based on the AI-based feedback. This relationship may have resulted from the growing expectations and trust among medical students regarding AI systems as future solutions for data organization and streamlined information access [68,69]. The data-driven nature of the feedback aligns more closely with such expectations compared to the improvement-focused feedforward. This also supports previous findings that attitudes toward and assumptions regarding new technologies like AI may serve as predictors of adoption [31,32]. Nevertheless, the lack of correlations between AI attitude and trustworthiness in general, as well as between AI attitude and the potential learning acceptance of feedforward, offers food for thought and highlights the need for further research in this area.

The limitations of our study include an uneven gender distribution and the “what if” design, in which the participants did not receive AI-based feedback and feedforward regarding their own performance but rather engaged in a hypothetical exercise. Additionally, the AI-based feedforward was supplementary to the previously presented AI-based feedback, and the *content* was thus linked. Furthermore, this study simulated a comprehensive fully automated AI-generated feedback and feedforward solution for simulated doctor–patient consultation videos that did not exist at the time of the study and was therefore partly based on manual work. This human element in the loop may have influenced our findings. Future studies could explore replacing this role with a large language model. However, a prototype of the NOVA interface which includes an automated debriefing function is currently under development [46] and could be potentially used as a basis for further evaluations. Finally, we utilized only a female avatar without controlling for other genders or morphological characteristics, such as the presence of facial hair or the position of the eyebrows [24,70].

## Conclusion

We conclude that medical students could be open to using a personalized and comprehensive automated AI-generated feedback and feedforward solution for simulated doctor-patient consultation in their personal medical communication training in the future. To comprehensively understand the implications of the reported findings for the future of AI in simulated doctor–patient consultation training, further research should be undertaken to provide students with fully AI-generated feedforward regarding their own consultation videos and to assess their perceptions of this feedforward.

## Supplementary Material

Supplementary material A.docx

## Data Availability

The datasets used and/or analyzed during the current study are available from the corresponding author on reasonable request.

## Endnotes

1. Integrated, competency-based medical curricula in Germany represent a contemporary approach to medical education, emphasizing a more patient-centered and practical learning experience. These curricula aim to equip medical students with a holistic understanding of healthcare by integrating biomedical (pre-clinical) basics, clinical knowledge, and skills, as well as research experience.

## Previous presentations

Bauermann, M., Schindler, A.-K., Hallmen, T., Kunz, M, André, E., Rotthoff, T. (2024). Studienprotokoll: “AI Effect—Untersuchung der lernwirksamen Annahme von KI-generierten und durch Avatare vermittelten Feedback und Feedforward zur ärztlichen Kommunikation bei Medizinstudierenden in einer Simulationsumgebung” [Study protocol: “AI Effect - Investigation of the learning-effective acceptance of AI-generated and avatar-mediated feedback and feedforward for medical communication from medical students in a simulation environment”]. In R. Bonvin (Ed.), Über Lernen, Lehren und Prüfen hinaus. der Mensch! Jahrestagung der Gesellschaft für Medizinische Ausbildung, August 5−9, 2024, Freiburg, Schweiz (pp. 115−117). German Medical Science GMS Publishing House. http://doi.org/10.3205/24gma145
