# Supplementary material for: Medical students’ perceptions of AI-based feedback and feedforward on communication skills in doctor–patient consultation - an acceptance study in a video-based simulation
Source: Med Educ Online. 2025 Dec 1;30(1):2592414. doi: 10.1080/10872981.2025.2592414 (PMC12671429; doi:10.1080/10872981.2025.2592414)
Supplement: Supplementary material — A.docx [file ZMEO_A_2592414_SM8861.docx]

Appendix – **AI-generated Feedback & Feedforward**

AI-Tool Analyses behavior student in the consultation video

| **annotated feature** | Value | feature description |
| --- | --- | --- |
| gaze | 89,0 % | Looking in the direction that was looked at the most (presumably into the eyes of the other person) |
| smile | 0., % | period in which smile was shown |
| sentiment | -0.18 | Connotation of the words used based on the transcript (distribution from -1 negative to +1 positive) |
| mutualgaze | 78.6 % | both conversation participants look in their respective main viewing direction |
| mutualsmile | 0,0 % | both participants show a smile both participants show a smile at the same time |
| headmovement | 7,0 % | head movements during the session |
| handmovement | 8,2 % | hand movements during the session |
| Segmentduration in seconds | 3.105 sec. | average duration of a spoken segment |
| wordcount per segment | 11.273 | Average number of words in a spoken segment |
| wordavglen in characters | 4.846 in characters | Average length of words in characters |
| wordpersec | 3.429 in words | Average spoken words per second |
| statement | 85,0 % | utterances that contained statements |
| question | 12,7 % | utterances that contained questions |
| Exlamation | 0,0 % | utterances that contained exclamations |

**Note.** The values measured by the Tool for the video stimulus shown; Percentages based on the duration of the session

**AI-generated Feedback**

I would now like to give you feedback on your conversational behavior based on my analyses. I would like to focus on the following three areas: your eye contact, your facial expressions and your speaking style.

You sought eye contact with your counterpart about 90% of the time during the entire conversation, with the proportion being 40% in the first two minutes, i. e. in the phase of opening the conversation.

Your facial expression seemed neutral to me throughout the conversation and there was no discernible smile.

I perceived your style of speech as consistently matter of fact, with only a few emotional expressions. You made mainly explanatory statements (86%) and asked questions and queries (14%). I perceived the proportion of speech between you and your counterpart as quite balanced with a slight preponderance of your speech.

**AI-generated Feedforward**

Based on my analyses, I suggest specific improvements in the areas of eye contact, facial expressions and speaking style.

With regard to eye contact, direct eye contact with your counterpart is recommended, especially when opening a conversation. This signals interest and conveys a feeling of appreciation. Aim for natural eye contact that is neither intrusive nor overly intense. Respect the personal boundaries of the person you are talking to. Avoid repeatedly looking at your watch to avoid giving the other person a feeling of disinterest. Instead, I recommend communicating the available time frame at the beginning of the conversation to structure the course of the conversation in advance.

An authentic smile is reflected in facial expressions and comes from the heart. Try to focus on the other person and their needs and make your smile appear spontaneous, genuine and appropriate for the situation. According to the literature, an authentic smile can be particularly helpful when opening a conversation.

Try to take the other person's emotions into account even more with your language. For example, you could say “I understand that you are worried, especially given the current situation. It is completely understandable and we will discuss the treatment together.” By using phrasing like this, you put the focus on the other person, which has been proven to increase acceptance and consent.

**Literature Base:**

First Paragraph:

Gorawara-Bhat, R., & Cook, M. A. (2011). Eye contact in patient-centered communication. *Patient Education and Counseling*, *82*(3), 442-447. <https://doi.org/10.1016/j.pec.2010.12.002>

Jongerius, C., Hillen, M. A., Romijn, J. A., Smets, E. M. A., & Koole, T. (2022). Physician gaze shifts in patient-physician interactions: Functions, accounts and responses. *Patient Education and Counseling*, *105*(7), 2116-2129. https://doi.org/10.1016/j.pec.2022.02.018

Second Paragraph

Krumhuber, E. G., Manstead, A. S. R., Cosker, D., Kappas, A., Marshall, D., & Rosin, P. L. (2007). Facial dynamics as indicators of trustworthiness and cooperative behavior. *Emotion*, *7*(4), 730-735. https://doi.org/10.1037/1528-3542.7.4.730
